# A Comparison of Two Species‐Discriminatory SNP Sets for Taxonomic Assignment in Hybridizing European White Oaks (*Quercus* sect. *Quercus*)

**DOI:** 10.1111/1755-0998.70221

**Published:** 2026-10-29

**Authors:** Christian Rellstab, Erwan Guichoux, Felix Gugerli, Antoine Kremer, Oliver Reutimann

**Affiliations:** ^1^ Swiss Federal Research Institute WSL Birmensdorf Switzerland; ^2^ INRAE, UMR1202 BIOGECO Cestas France; ^3^ Université de Bordeaux, UMR1202 BIOGECO Talence France; ^4^ Institute of Integrative Biology, ETH Zurich Zurich Switzerland

**Keywords:** admixture, ascertainment bias, hybridization, *Quercus*, species identification, white oaks

## Abstract

Species identification is essential for conservation, forest management, and understanding ecological and evolutionary dynamics, such as adaptive introgression and maintenance of species boundaries in hybridizing taxa. Species‐discriminatory marker sets have been shown to be more accurate than morphological identification, especially in hybridizing species complexes, and provide a more affordable alternative to genome‐wide analyses. However, to our knowledge, no species complex exists for which several marker sets are available, let alone have been compared to each other. Moreover, it remains unclear how well marker sets can be applied to additional, closely related species. Here, we compared two independently developed SNP sets, one derived from amplicon sequencing and one from whole‐genome sequencing, for three morphologically similar and hybridizing European white oak species *sensu lato* ( *Quercus robur* , *Q. petraea* , and *Q. pubescens*). We applied the SNP sets to 560 samples, including samples of known pure ancestry, controlled crosses, natural populations, and four additional white oak species. Congruency between SNP sets was very high, but species assignment was slightly more accurate for the reference samples used in the development of the respective SNP set. Ancestry coefficients for first‐generation hybrids were more precise with the SNP set based on whole‐genome data. In a natural population, there was limited congruency between SNP sets in quantifying admixture. Notably, the SNP sets were able to identify up to two additional white oak species. Our study highlights the great potential of species‐discriminatory SNP sets in science and practice, while demonstrating that ascertainment bias should not be neglected, and accurate introgression assessments may require genome‐wide analyses.

## 1 Introduction

Accurate species identification is fundamental in both science and practice, as it underpins our understanding of ecological and evolutionary processes and supports decision making in, e.g., biodiversity conservation and forest management (Mace 2004). Molecular genetic approaches, from early microsatellites (nuclear simple sequence repeats, nSSRs, e.g., Guichoux et al. 2011) over DNA barcoding (Hebert et al. 2003) to whole‐genome sequencing (Gan et al. 2024), have proven to be a great aid for species identification, especially in species groups that are difficult to distinguish based on morphology alone (e.g., Baiakhmetov et al. 2021). Additionally, genetic markers and analytical tools allow the reliable identification of hybrids and, thus, the quantification of admixture and introgression (e.g., Oliveira et al. 2015, reviewed in Twyford and Ennos 2012). This is particularly relevant because hybridization is widespread in nature, especially among plants, where at least 25% of species hybridize (Mallet 2005). Hybridization can profoundly influence evolutionary outcomes, for example by increasing genetic diversity (Guo et al. 2025) or facilitating adaptive introgression (Suarez‐Gonzalez et al. 2018).

For molecular species assignment and admixture assessment, whole‐genome sequencing is likely excessive, more costly, and more computationally resource intensive compared to targeted sequencing/genotyping approaches, particularly for species with large and complex genomes. This is especially true for applied analyses, where financial resources and analytical know‐how are often limited. Consequently, species‐diagnostic SNP panels have become widely adopted (e.g., Chang et al. 2022; DeHaan et al. 2014; Hagen et al. 2026; Hohenlohe et al. 2011; Marques et al. 2017), providing species assignment with high resolution at limited costs. Furthermore, diagnostic marker approaches do not necessarily require reference genome assemblies, making them applicable to many species and taxonomic groups.

The development of species‐discriminatory SNP sets presents several challenges, including marker discovery, minimizing ascertainment bias and maximizing taxonomic resolution. First, the discovery of candidate SNPs ideally exploits genome‐wide data (Geibel et al. 2021), which are often unavailable, hence necessitating the use of alternative datasets. Second, one needs a reliable set of reference samples consisting of pure individuals of known taxonomy, which can be difficult to obtain for morphologically similar and/or hybridizing species. Third, to ensure broad applicability, reference samples should be representative of species' geographic distributions, covering as much of a species' genetic diversity as possible. Fourth, samples of additional closely related species not included in the discovery panel may be misidentified as one of the species in the discovery panel or—depending on the assignment method—even bias the assignment of the target species. Finally, if accurate quantification of admixture is an important goal of a study, markers should ideally be validated with admixed individuals of known co‐ancestry.

In white oaks (*Quercus* sect. *Quercus*), widespread hybridization obscures species boundaries, making morphological species identification particularly challenging (Kremer and Hipp 2020). These ecologically, economically, and culturally important tree species primarily occur at low to intermediate latitudes across the Northern hemisphere. In temperate forests of Central Europe, three white oak species are frequently present: *Q. robur* L., *Q. petraea* (Matt.) Liebl., and *Q. pubescens* Willd. (Eaton et al. 2016; Pasta et al. 2016). Although they generally occupy different ecological niches (e.g., Leroy et al. 2020; Rellstab, Zoller, et al. 2016), they often co‐occur in mixed forests (Curtu et al. 2009; Salvini et al. 2009), with their fine‐scale spatial arrangement largely determined by the microhabitat (Reutimann et al. 2023; Truffaut et al. 2017; Zimmermann et al. 2025). Traditionally, these species have been differentiated based on leaf morphology (Dupouey and Badeau 1993; Gugerli et al. 2007; Kremer et al. 2002; Rellstab, Bühler, et al. 2016), but leaf traits were found to be highly overlapping among the species, especially between *Q. petraea* and *Q. pubescens*, the most closely related pair of the three species (Hipp et al. 2020; Leroy et al. 2017). Besides phenotypic plasticity and weak genetic differentiation among the species, genetic admixture is most likely contributing to morphological overlap among trees. The three species frequently hybridize (especially *Q. petraea* and *Q. pubescens*) and populations with various levels of admixture (later‐generation hybrids and backcrosses) are common (e.g., Degen et al. 2023; Lepais et al. 2009; Pakull et al. 2025; Reutimann et al. 2023). Still, the parental species largely retain their species integrity (e.g., Gailing and Curtu 2014; Leroy et al. 2020; Reutimann et al. 2020).

To overcome uncertainties with morphological species assignment, various sets of genetic markers have been developed for the white oak species complex (Degen et al. 2021; Guichoux et al. 2011; Kremer et al. 2024; Neophytou 2014; Reutimann et al. 2020; Schroeder and Kersten 2023) and were used to investigate, e.g., introgression and admixture (Degen et al. 2023; Lepais et al. 2009; Reutimann et al. 2020) or regional and local drivers of taxon distribution (Neophytou and Michiels 2013; Reutimann et al. 2023; Zimmermann et al. 2025). Note that commonly applied species barcode markers, usually based on chloroplast DNA (cpDNA), are not suitable for species discrimination in white oaks because species widely share cpDNA haplotypes (Petit et al. 1993, 2002). Moreover, uniparental markers are generally not informative regarding levels of admixture. Genetic marker sets developed for white oaks have been shown to outperform morphological assessments (Rellstab, Bühler, et al. 2016), and SNP sets outperform nSSRs in species discrimination (Reutimann et al. 2020). However, a direct comparison of different SNP sets, developed by different research groups using different reference individuals and genomic resources, remains unexplored. To our knowledge, such complementary diagnostic SNP sets do not exist for any species complex nor have different sets been systematically compared. Moreover, it remains unknown how such SNP sets perform when applied to closely related species that were not included in the reference panel.

Here, using European white oaks as a case study, we take advantage of the unique situation in which two different species‐discriminatory SNP sets were developed for the same hybridizing species complex. This allows us to address the following questions: (1) Are the species assignments of reference samples congruent using the two independently developed discriminatory SNP sets? (2) Can the two SNP sets accurately assign F1 hybrids from controlled crosses and yield congruent taxon assignments in natural admixed populations? (3) To what extent are the developed SNP sets effective for distinguishing additional white oak species and F1 hybrids that were not included in the reference panel? Our results refine existing knowledge of white oak species delimitation and admixture, while offering broadly applicable insights for future development of species‐discriminatory SNP sets in other taxonomic groups in respect to, e.g., available genomic resources, ascertainment bias, and non‐target species.

## 2 Material and Methods

### 2.1 Species‐Discriminatory SNP Sets

Throughout the manuscript, we will refer to the original target species ( *Q. robur* , *Q. petraea* , and *Q. pubescens*) as ROB, PET, and PUB, respectively. It is important to note that we consider these three species as collective groups ( *Q. robur* *s*.*l*., *Q. petraea* *s*.*l*., *and Q*. pubescens *s*.*l*.), thereby pooling the various putative subspecies that have been described in the literature for the species complex. These subspecies often show distinct morphological characteristics but have rarely been confirmed by genetic analyses (examples for PET and PUB in Italy: Di Pietro et al. 2021, 2026). We therefore acknowledge that our dataset might include some of the known subspecies, especially in the southern and eastern regions of our sampling range.

We used two different species‐discriminatory SNP sets to genotype samples (for an overview, see Table S1). One SNP set (58 biallelic SNPs developed at the Swiss Federal Research Institute WSL, Reutimann et al. 2020), hereafter referred to as KASP SNP set, uses the Kompetitive Allele Specific PCR (KASP, LGC Genomics, Hoddesdon, UK, Semagn et al. 2014) genotyping technology. It has been shown to reliably differentiate between ROB, PET, and PUB, although none of the alleles at the 58 loci was found to be fixed for a species, and was used to assess admixture levels in white oaks (Reutimann et al. 2020, 2023; Zimmermann et al. 2025). The second SNP set (38 SNPs established at INRAE, Kremer et al. 2024), hereafter referred to as MassARRAY SNP set, uses the MassARRAY genotyping platform (Agena Bioscience, San Diego, USA, Jurinke et al. 2002) and was developed to assign four white oak species (additionally including *Q. pyrenaica* [PYR]) based on a candidate set of 61 SNPs. Here, we used 33 markers of the final and five from the remaining candidate SNP set of Kremer et al. (2024) for genotyping (Table S2).

The two SNP sets compared in this study were developed following two different strategies (Table S1). The SNPs of the KASP SNP set were identified based on morphological assignment information from 71 populations of ROB, PET, and PUB in Switzerland (Rellstab, Bühler, et al. 2016) combined with 3576 SNPs from a targeted and pooled amplicon sequencing approach of the same populations (Rellstab, Zoller, et al. 2016). Additionally, the discovery panel included 16 European‐wide reference samples for the three species. The MassARRAY SNP set contains 7–12 near‐diagnostic markers for each ROB, PET, PUB, and PYR. Near‐diagnostic SNP markers of a given species were defined as SNPs with one allele close to full fixation in this species and almost absent in the remaining three species (Kremer et al. 2024). The MassARRAY SNP set originated from a screening of whole‐genome pooled sequences of ROB, PET, PUB, and PYR populations (about 20 individuals per species, Leroy et al. 2020) from the Southwest of France and of an individual capture‐based sequencing method in a mixed ROB/PET population from the Northwest of France (Lesur et al. 2018). Kremer et al. (2024) further tested for the maintenance of near‐diagnostic allele frequencies across a larger sample of populations throughout the total distribution range of the species. In summary, the discovery strategies for discriminating oak species differed between the two marker systems in how interspecific genomic variation was sampled and analysed. The KASP markers resulted from a multi‐locus strategy based on a reduced exploration of genomic variation, while the MassARRAY focused on single‐locus species differentiation based on whole‐genome exploration.

### 2.2 Specimens and Genotypes

Genotypic information for the SNP set‐specific reference samples of the common three species (ROB, PET, PUB) were obtained from Reutimann et al. (2020) and Kremer et al. (2024). For the KASP SNP set, ‘reference samples’ here refer to the training set used in Reutimann et al. (2020), i.e., the samples that were used for SNP discovery and are not genetically admixed. We will refer to them as WSL reference samples. For MassARRAY, ‘reference samples’ are samples from across the distribution range (see above) that were not admixed after genetic assignment analyses in Kremer et al. (2024). We will refer to them as INRAE reference samples. Reference trees for both SNP sets were assigned morphologically by expert knowledge (authors or collaborators) or, in some cases, using unsupervised clustering methods with genetic data only or a combination of genetic and morphological data (e.g., Rellstab, Bühler, et al. 2016; Reutimann et al. 2020).

For this study, we additionally genotyped each set of reference samples with the complementary SNP set (WSL samples with MassARRAY, INRAE samples with KASP). Additionally, we used both SNP sets to genotype six controlled crosses (F1 hybrids) representing all three species combinations (Kremer et al. 2024), with two crosses per combination. Moreover, we obtained DNA of the EvolTree Intensive Study Site (ISS) Valais (Switzerland, 46°18′ N/7°40′ E, consisting of PET and PUB) from the EvolTree DNA repository (Stierschneider et al. 2016) and genotyped these samples with both SNP sets. Intensive Study Sites are regional forests selected for monitoring genetic diversity and studying genetic processes (gene flow, hybridization, etc.) within the EvolTree research network (https://www.evoltree.eu/), where sets of trees are georeferenced and genotyped (Lefèvre et al. 2016).

Beyond the three focal taxa, we included samples from additional white oak species: *Q. pyrenaica* (PYR) from Kremer et al. (2024), *Q. frainetto* (FRA), *Q. canariensis* (CAN), *Q. faginea* (FAG), hybrids of *Q. faginea* and *Q. pyrenaica* (FAG×PYR) obtained through collaborators, and FRA collected through our own sampling activity in Greece. For PYR specifically, we also included, for each combination with ROB, PET, and PUB, two controlled crosses (F1) from Kremer et al. (2024) and a selection of samples from an ISS population (Landes, France, 44°13′ N/1°05′ W) where both ROB and PYR are present. These samples were also genotyped with both SNP sets.

In total, we analysed 560 samples with less than 10% missing data, 559 with KASP, 379 with MassARRAY, and 378 on both platforms, representing 313 reference samples (including 28 PYR), 12 controlled crosses between ROB/PET/PUB/PYR, 34 samples from ISS Valais (PET and PUB), 16 from ISS Landes (ROB and PYR), and 184 from additional taxa (excluding PYR, Table S3). For samples collected specifically for this study, DNA extraction of silica gel‐dried leaves was done with an oak‐specific sbeadex maxi plant kit (LGC Genomics, Berlin, Germany) using a Kingfisher 96 Flex (Thermo Fisher Scientific, Waltham, MA, USA).

### 2.3 Sample Sets

Each of the two discriminatory SNP sets (KASP and MassARRAY) was developed based on its own reference sets (WSL and INRAE) of the three and four *Quercus* species, respectively, with no overlap between reference sets. To assess congruency among species assignment (question 1), we evaluated the assignment of each reference sample set (WSL and INRAE) with both SNP sets (KASP and MassARRAY). To do so, we created sample sets 1 (WSL) and 2 (INRAE, without PYR), each containing only samples that were successfully genotyped on both platforms (Table S4). To address the accuracy in assigning hybrids (question 2), we tested if the six PET/PUB/ROB F1 hybrids from controlled crosses and samples from a mixed natural population (PET and PUB, ISS Valais) can be accurately assigned and/or lead to similar results with both SNP sets. For this, we created two additional sample sets 3 and 4 (Table S4), each containing all reference samples of the respective SNP set (not only those that were successfully genotyped on both platforms) and the crosses/ISS samples. To assess the ability of the two SNP sets to identify other white oak species (question 3), we first created sample sets for KASP and MassARRAY (sets 5 and 6, Table S5) containing their original reference samples and the additional taxa (PYR, CAN, FAG, FAG×PYR, and FRA). To test the assignment of F1 hybrids (available for ROB, PET, PUB, and PYR) and ISS Landes samples (ROB and PYR), we then created sample sets 7 and 8 (Table S5) containing the respective reference samples and the hybrids/ISS samples. Because the WSL reference sample set does not originally include PYR samples, we used the PYR reference samples from the INRAE reference set genotyped with KASP in the KASP reference set.

### 2.4 Species Assignment

For the assignment of the reference samples (question 1, sample sets 1 and 2) and additional taxa (question 3, sample sets 5 and 6), we used a standard and ‘naïve’ approach in structure 2.3.3 (Pritchard et al. 2000) with admixture model and correlated allele frequencies to group individuals of different sample sets into genetic clusters using the two above defined sets of SNPs. The number of clusters (*K*) was set to 1–10 for sample sets 1 and 2 and 1–15 for sample sets 5 and 6. We ran 10 iterations for each level of K and 1,000,000 repetitions after a burn‐in period of 100,000 runs. Runs were summarized with structure harvester 0.6.93 (Earl and vonHoldt 2012). We report the mean and standard deviation of posterior probability of all 10 iterations and show the co‐ancestry coefficients (*Q*) re‐ordered and averaged by clumpak (Kopelman et al. 2015). As structure outputs continuous *Q*‐values, we also classified each sample as ‘pure’ species if the maximum *Q*‐value was equal or above 0.9 (as recommended by Vähä and Primmer 2006). All individuals with a maximum *Q*‐value of less than 0.9 were classified as admixed (ADM).

For the assignment of F1 hybrids from controlled crosses and natural populations (questions 2 and 3), we used structure with the USEPOPINFO option to define training (ROB, PET, and PUB for sample sets 3, 4, 7, and 8; additionally PYR reference samples for sample sets 7 and 8) and test samples (all others). This model assumes that the training set (i.e., reference samples) is correct and clusters the test individuals based on predefined clusters from training individuals. The resulting *Q*‐matrix contains estimates of cluster ancestry coefficients for training individuals and assignment probabilities for test individuals. Except for the USEPOPINFO parameter, all settings were equal to the standard structure runs. We analysed only the K that corresponds to the expected number of species (i.e., *K* = 3 for sample sets 3 and 4, *K* = 4 for sample sets 7 and 8). Since clumpak cannot handle the output format of structure with USEPOPINFO, we selected the run with the highest posterior probability for further analysis. To compare the *Q*‐values resulting from different SNP sets in the ISS populations, we performed linear regressions of *Q*‐values of the test individuals.

For the analysis of reference samples and the ISS Valais population, we performed another, assumption‐free and non‐clustering approach for species discrimination, i.e., principal component analysis (PCA). We imported the structure input files into R using adegent (Jombart 2008 ), imputed missing data with mean values using poppr (Kamvar et al. 2014) and computed the PCA with ade4 (Chessel et al. 2004), retaining the first two principal components (PCs). To quantify the ability of the marker/reference sample sets to separate the reference samples of the three species, we calculated the separation/cohesion ratio according to Davies and Bouldin (1979) using the coordinates of the first two PCs. Separation (among clusters, i.e., species) was calculated as the average distance between the centroids of each cluster. Cohesion (within clusters) was assessed by using the average distance (weighted by cluster size) of each point to the centroid of its cluster. A high separation/cohesion ratio indicates that the distance between clusters is large and/or the variation within clusters is small, leading to a good separation of the species. For the ISS Valais populations, we tested for congruency among the two SNP sets using linear regression of the PC coordinates of the test individuals.

### 2.5 Genetic Diversity and Differentiation

To compare genetic diversity estimates between the two genotyping approaches and characterize the reference sample sets, we extracted pairwise values of genetic differentiation (net nucleotide distance, *D*) among clusters from the structure output of the analysis of the reference samples. This measure represents the average probability that a pair of alleles is different, with *D* = 0 for identical individuals (Pritchard et al. 2000). Moreover, we estimated expected heterozygosity (*H*_e_) from each cluster. All these values were extracted from the run with the highest posterior probability for the K of interest.

## 3 Results

### 3.1 Assignment of Reference Samples

In the analysis of WSL reference samples with both KASP and MassARRAY SNP sets, the posterior probability of *K* reached a plateau for *K* = 3, clearly separating the three white oak species (Figure 1a,b). Both SNP sets reliably assigned samples to the correct species corresponding to the WSL reference set, except for two PET individuals (containing a substantial amount of PUB [38% and 27%]) when using MassARRAY. These samples originate from Swiss populations in the western Jura range (Bevaix) and the western Valais (Vionnaz), respectively. Moreover, there was a slightly higher level of admixture with PET in PUB when using MassARRAY (average 5%) than when using KASP (average 2%). The *Q*‐values of KASP and MassARRAY mostly corresponded to each other, but MassARRAY *Q*‐values showed larger variation than KASP *Q*‐values (Figure S1). This led to the assignment of some WSL reference samples into the admixed (ADM) category by MassARRAY, especially for PUB samples (Figure S2). However, no trees were assigned to a different pure species using the MassARRAY SNP sets.

**FIGURE 1:**
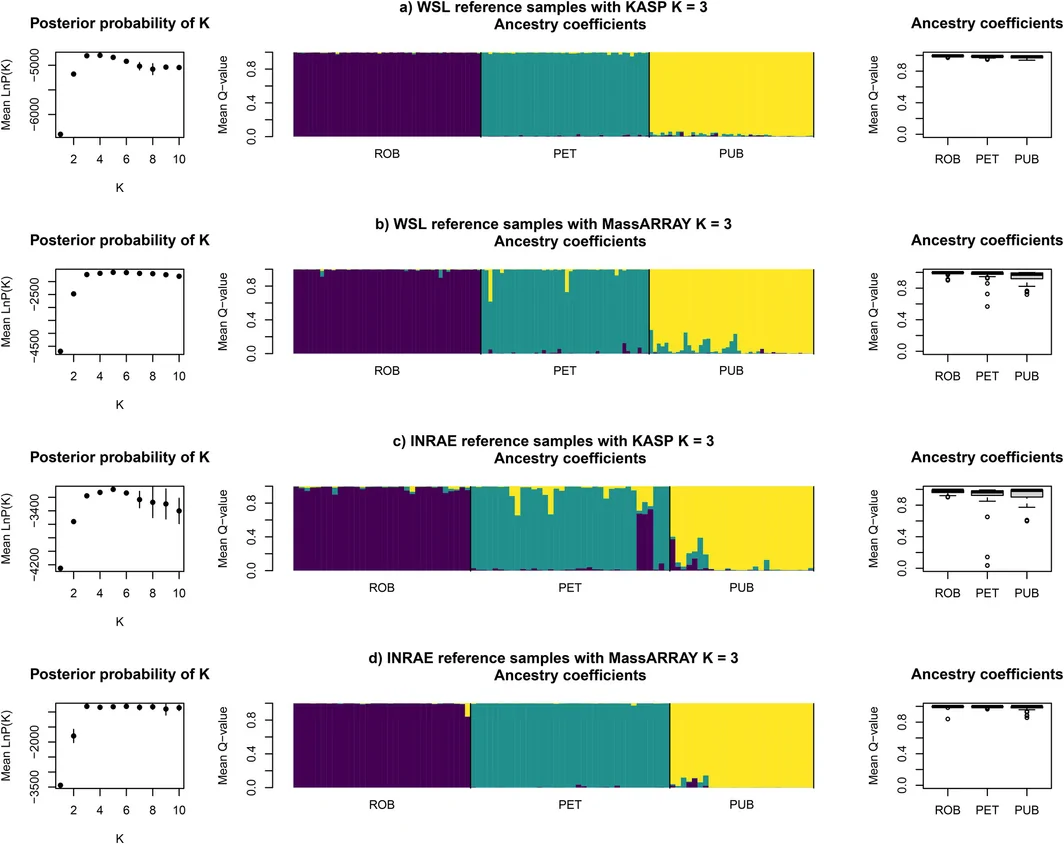
structure analyses of the *Quercus* spp. reference samples using the WSL reference samples with the (a) KASP and (b) MassARRAY SNP set, and the INRAE reference samples with the (c) KASP and (d) MassARRAY SNP set. The KASP SNP set was developed based on WSL reference samples, whereas the MassARRAY SNP set was derived from INRAE reference samples. Left: Mean posterior probability across 10 replicate runs (LnP[*K*]) and its standard deviation for each number of clusters (*K*). Centre: Bar plots of mean ancestry coefficients (*Q*‐values) for each sample at *K* = 3. Right: Boxplots of mean ancestry coefficients (*Q*‐values) of the three species. ROB = *Q. robur* , PET = *Q. petraea* , PUB = *Q. pubescens*.

The analysis of the INRAE reference samples led to similar results (Figure 1c,d) as with the WSL reference samples. The posterior probability of *K* plateaued at *K* = 3, with slightly higher probability using KASP markers for *K* = 4–6. Discrepancies between KASP and MassARRAY were more pronounced than in the analysis of the WSL reference samples. Interestingly, KASP SNPs assigned three PET reference individuals from Tronçais (France) to ROB‐like trees (67%–72% ROB assignment, Figure 1c). Moreover, more admixture between PET and PUB was present using the KASP SNP set, as seen in the comparison of *Q*‐values of the two marker sets (Figure S1) and in the assignment into ‘pure’ species (Figure S2). In the latter analysis, a substantial part of PET (22%) and PUB (23%) were classified as admixed. However, again, no assignment of samples to one of the alternative species was observed.

The PCAs showed that MassARRAY exhibited the strongest ability to separate the three species in both the WSL and INRAE reference sample sets, expressed as the highest separation to cohesion ratios (Figure 2). However, in the case of the WSL reference sample set, this was mainly driven by the strong separation of ROB from the other two species, whereas the separation between PET and PUB was similar in both reference sets. Using KASP for the INRAE reference set showed the lowest separation.

**FIGURE 2:**
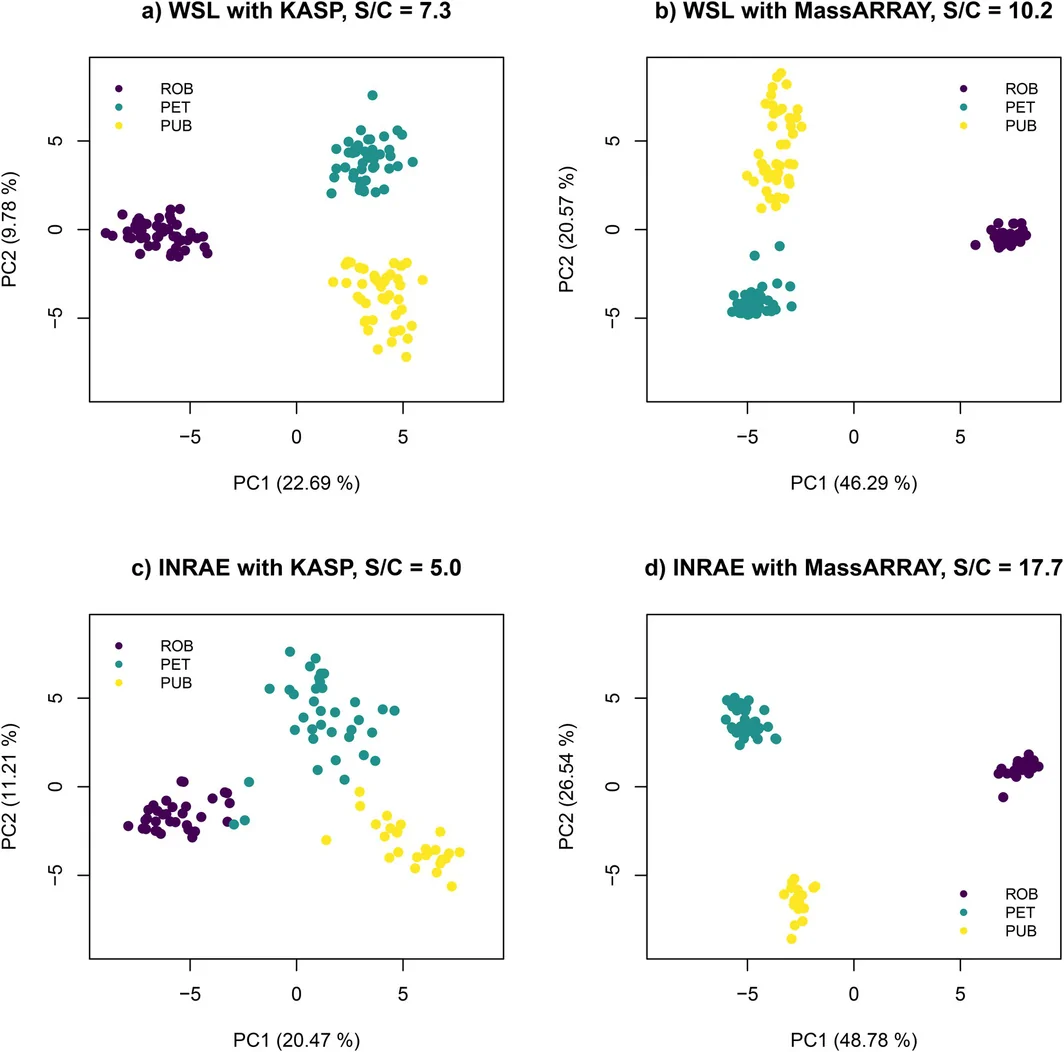
Taxonomic separation of *Quercus* spp. individuals using principal component analyses (PCAs) of the WSL (top, a,b) and INRAE (bottom, c,d) reference samples genotyped with KASP (left, a,c) and MassARRAY (right, b,d). The KASP SNP set was developed based on the WSL reference samples, whereas the MassARRAY SNP set was derived from the INRAE reference samples. S/C indicates the separation/cohesion ratio, a measure of how well the approach can separate the three a priori categorized species. ROB = *Q. robur* , PET = *Q. petraea* , PUB = *Q. pubescens*.

The MassARRAY SNP set showed stronger genetic differentiation (*D*) between clusters (i.e., species) and lower within‐cluster genetic diversity (*H*_e_) than the KASP SNP set (Figure S3). Generally, and especially with MassARRAY (*D* = 0.39–0.47), ROB was the most differentiated species, whereas the other two species did not differ that much (*D* = 0.21 for MassARRAY). Whereas PUB showed the highest *H*_e_ in the WSL reference samples (0.16 and 0.21 for KASP and MassARRAY, respectively), it had the lowest *H*_e_ in the INRAE reference sample set (0.14 and 0.03 for KASP and MassARRAY, respectively), for which  PET showed the highest *H*_e_ in both SNP sets (0.20 and 0.08 for KASP and MassARRAY, respectively, Figure S3c,d).

### 3.2 Assignment of F1 Hybrids and a Mixed Natural Population

Using the USEPOPINFO option in structure and the respective reference samples as training samples, the assignment of F1 hybrids from controlled crosses resulted in nearly a 50:50 ratio of the parental species, except for one PETxPUB hybrid (QS1*Pu1.1) with KASP (Figure 3a,c). In all cases, there was a small, negligible assignment proportion of the third species present. The KASP method with WSL reference samples generally led to slightly higher proportions of PUB than the MassARRAY method with INRAE reference samples.

**FIGURE 3:**
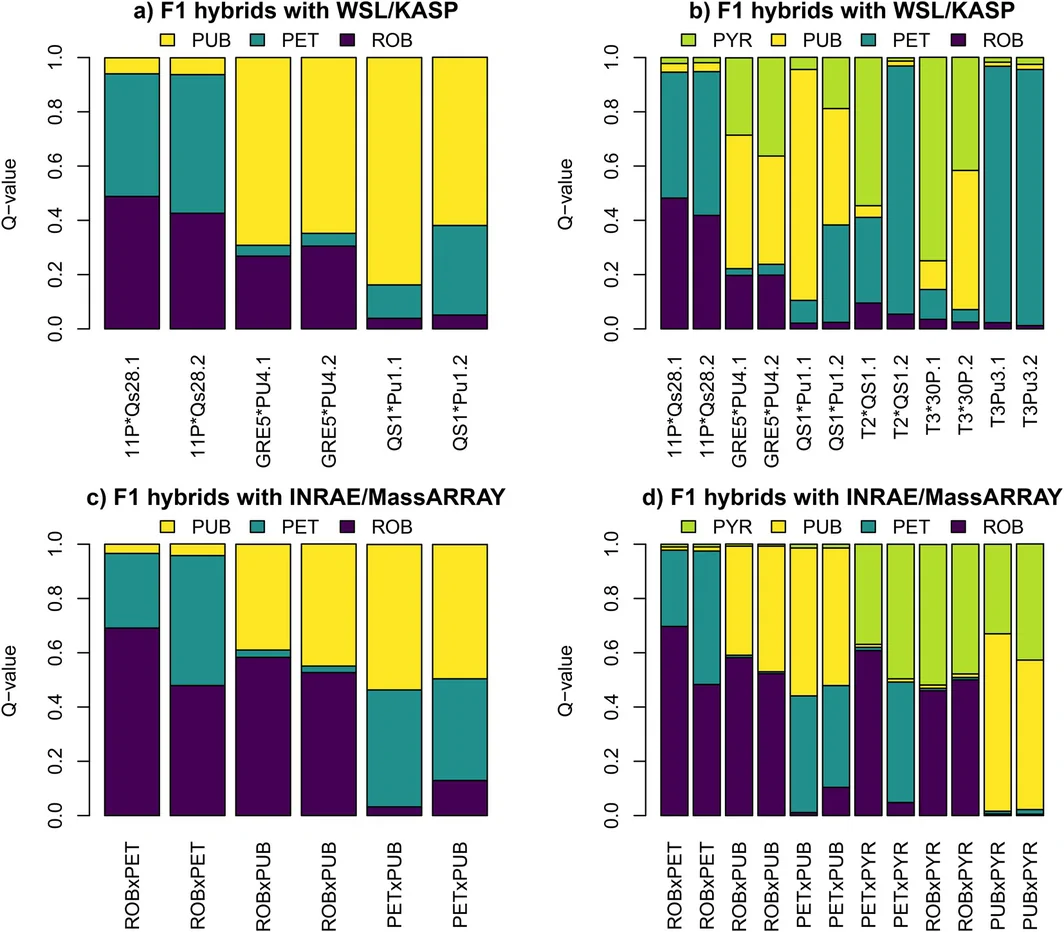
Taxon assignment of F1 hybrids in *Quercus* spp. Results from structure USEPOPINFO (*K* = 3) analyses using (a) the KASP SNP set and WSL reference samples as training samples and (c) the MassARRAY SNP set with INRAE reference samples as training samples; and from structure USEPOPINFO (*K* = 4) analyses using (b) the KASP SNP set and WSL reference samples as training samples (except for PYR, where the INRAE reference samples were used) and (d) the MassARRAY SNP with INRAE reference samples as training samples. Samples are labelled with their original name in the upper, and with the parental species of the F1 hybrids in the lower panels. ROB = *Q. robur* , PET = *Q. petraea* , PUB = *Q. pubescens*, PYR = *Q. pyrenaica*.

The assignment congruency between the two SNP sets of the mixed PET/PUB ISS Valais population, however, was rather low (Figure 4a,b). Although the general trend of the regression between assignment probabilities was positive (slope = 0.22 for PET and slope = 0.17 for PUB), they were not statistically significant (*p* = 0.11 for PET and *p* = 0.16 for PUB). Again, the WSL/KASP method assigned higher proportions of PUB than the INRAE/MassARRAY method. The PCA for the same dataset showed a similar result; PC coordinates of WSL/KASP were not significantly correlated with those of INRAE/MassARRAY (Figure S4, top row). To further explore possible reasons for the low agreement between the two assignment approaches for this population, we examined various datasets (different reference samples, analyses without ROB) with additional PCAs (Figures S4 and S5); the linear regressions of assignment probabilities were not significant, except for using INRAE reference samples from all three species (Figure S4, third row). Note that we assessed only the agreement of the PCs exhibiting the best PET/PUB differentiation, ignoring other PCs that may contribute additional information on species discrimination.

**FIGURE 4:**
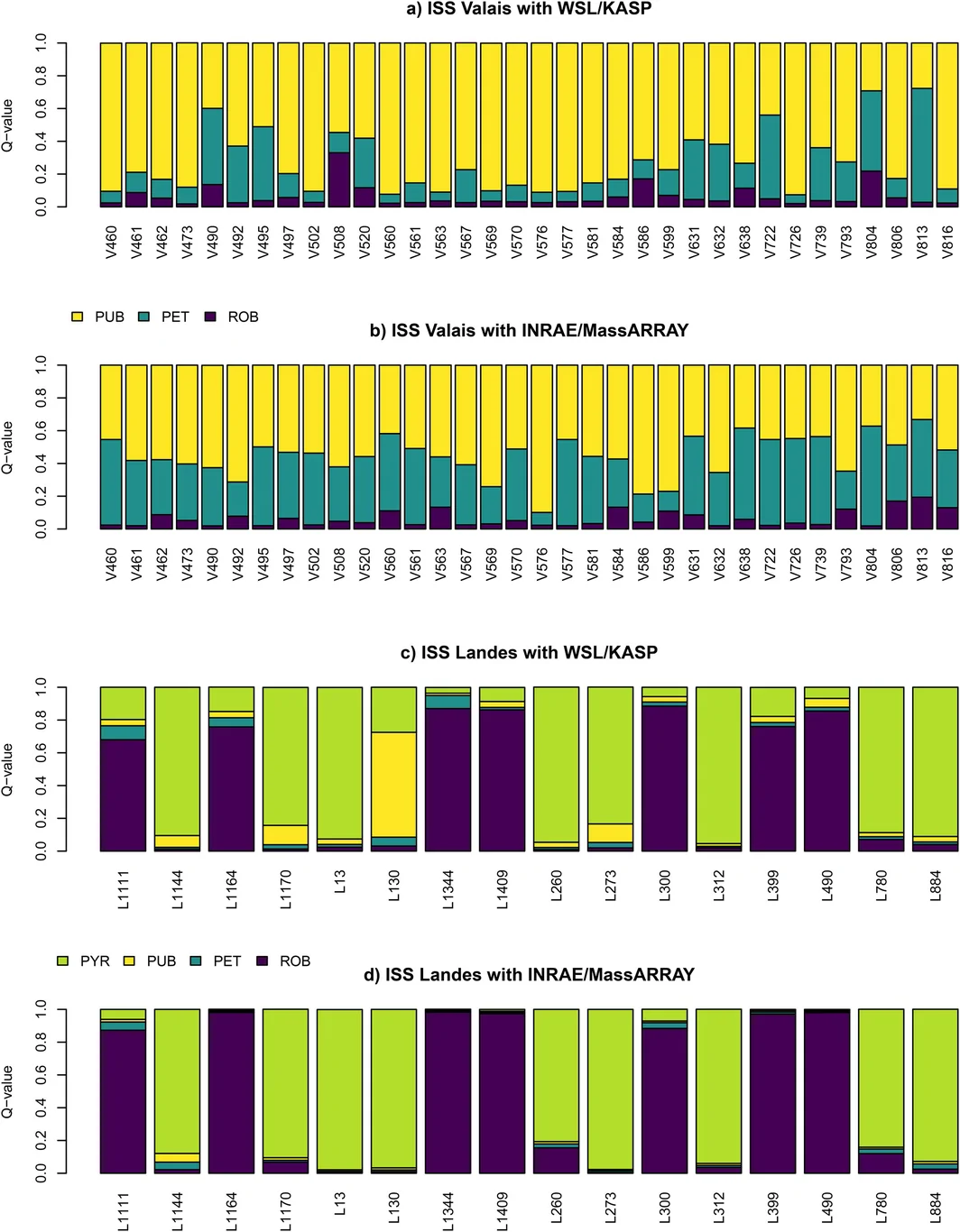
Taxon assignment of the ISS Valais and ISS Landes populations of *Quercus* spp. Upper part: Results from structure USEPOPINFO (*K* = 3) analyses for ISS Valais using (a) KASP and WSL training samples; (b) MassARRAY with INRAE training samples. Lower part: Results from Structure USEPOPINFO (*K* = 4) analyses using (c) KASP and WSL reference samples as training samples (except for PYR, where the INRAE reference samples were used) and (d) MassARRAY with INRAE reference samples as training samples. ROB = *Q. robur* , PET = *Q. petraea* , PUB = *Q. pubescens*, PYR = *Q. pyrenaica*.

### 3.3 Assignment of Additional Taxa

Based on the KASP SNP set and the WSL reference samples of ROB, PET, and PUB, the posterior probability of *K* was highest at *K* = 6 (Figure 5a) for the analysis with additional taxa. In the hierarchical development with increasing *K* (Figure S6), *K* = 2 did not only separate ROB, but also PET from the rest. FRA was assigned to an own cluster with *K* = 4 before the clear separation of PUB and PYR, which is only visible at *K* = 6. No additional new clusters could be assigned to species with *K* values above 6. With *K* = 6, the KASP SNP set could therefore discriminate between all species but CAN and FAG (Figure 5a). Some individuals of PUB and PYR showed substantial admixture with PET and CAN/FAG, respectively. Some, but not all individuals of the FAG×PYR group showed an admixed pattern of CAN/FAG and PYR. Moreover, there seemed to be a few potentially wrongly sampled trees in FAG and FRA. In summary, the KASP dataset could assign two additional species (PYR and FRA) than it was initially developed for.

**FIGURE 5:**
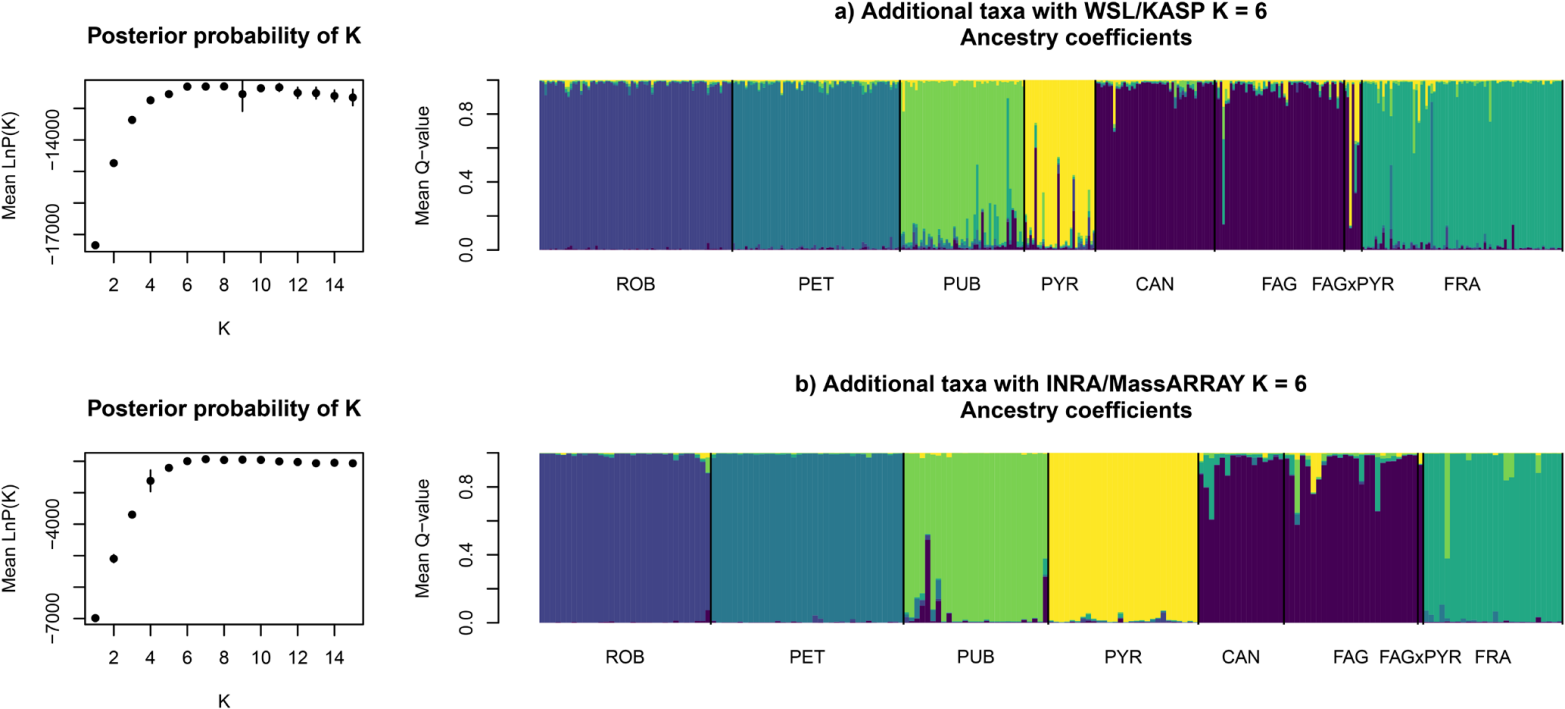
structure analysis of additional Quercus taxa using the (a) KASP SNP set with WSL reference samples for ROB, PET, and PUB, and INRAE reference samples for PYR and (b) MassARRAY SNP set with INRAE reference samples for ROB, PET, PUB, and PYR. Left: Mean posterior probability across 10 replicate runs (LnP[*K*]) and its standard deviation for each number of clusters (*K*). Right: Bar plots showing the mean ancestry coefficients (*Q*‐values) for each tree at *K* = 6. For bar plots showing other values of *K*, see Figures S6 and S7. ROB = *Q. robur* , PET = *Q. petraea* , PUB = *Q. pubescens*, PYR = *Q. pyrenaica*, CAN = *Q. canariensis*, FAG = *Q. faginea*, FRA = *Q. frainetto* .

With MassARRAY and the INRAE reference sample set for ROB, PET, PUB, and PYR, the posterior probability for *K* reached a plateau at around *K* = 7 (Figure 5b). *K* = 2 separated ROB from the rest, *K* = 3 introduced PYR, *K* = 4 PET, *K* = 5 PUB, and *K* = 6 FRA (Figure S7). At higher *K*, no additional cluster could be clearly assigned to an additional species, leaving CAN and FAG unassigned, although at *K* = 7, the new cluster was only present in FAG in admixture with CAN. FAG×PYR hybrids belonged to the same cluster as CAN and FAG in *K* = 6. In summary, the MassARRAY SNP set could assign one more species (FRA) than it was developed for and inferred generally less admixture than the KASP dataset.

Since KASP markers using WSL training samples generally assigned PYR trees correctly (Figure 5a), we also tested the assignment of F1 hybrids involving PYR from controlled crosses and a mixed natural population that included ROB and PYR (ISS Landes). MassARRAY quite accurately identified the expected 50:50 ratio in F1 hybrids, except for T2*QS1.1, which was identified as ROB×PYR instead of PET×PYR (Figure 3d). This was different with the KASP SNP set that sometimes failed to identify hybrids in cases where PYR was involved and even in one case where PYR was not involved (QS1*Pu1.1, Figure 3b). Interestingly, unlike the MassARRAY SNP set, it identified the correct hybrid nature of T2*QS1.1. Also in this analysis, KASP generally identified more PUB proportions than MassARRAY.

In contrast to the analysis in the ISS Valais population, the agreement between the two SNP sets was very high in the French ISS population from Landes (linear regressions, slope = 1.14 and slope = 1.02 for PYR and ROB, respectively, *p* < 0.001, Figure 4c,d), except for tree L130 that was mainly assigned to PUB instead of PYR using the KASP SNP set. Generally, the trees only showed low to almost no admixture in this population.

## 4 Discussion

Genetic approaches offer an invaluable alternative to morphological assessments for taxon assignment in closely related, morphologically similar, and hybridizing species. Unlike morphological methods, genetic approaches are not affected by plastic or overlapping trait characteristics and can provide more accurate taxonomic assignment and estimates of admixture levels. Species‐discriminatory SNP sets are generally more affordable and less complex than genome‐wide approaches but come with a possible trade‐off of lower taxonomic resolution (but see, e.g., Hagen et al. 2026) and ascertainment bias. Here, we took advantage of two independently developed SNP sets (Kremer et al. 2024; Reutimann et al. 2020) to identify several white oak species *sensu lato* (*Quercus* section *Quercus*) and their admixed individuals. By genotyping hundreds of individuals with the two species‐discriminatory SNP sets, we showed that both approaches lead to highly congruent species assignments, but each SNP set was best performing on their respective reference sample set. Accuracy and congruency of both SNP sets in identifying F1 hybrids from controlled crosses were generally high, but lower in an admixed natural population. Finally, both SNP sets were able to identify additional white oak taxa that were not included in the SNP design, while they showed limitations in identifying F1 hybrids involving additional taxa. Together, our results illustrate not only the strengths and limitations of SNP‐based species discrimination in a complex and hybridizing plant group but also provide guidance for applying similar tools in other taxonomically challenging systems.

### 4.1 Discriminatory Power and Congruency of the Two Species‐Discriminatory SNP Sets

Analysis of genetic differentiation among species and diversity within species showed that the MassARRAY SNP set led to stronger differentiation (*D*) and less within‐cluster diversity (*H*_*e*_) than the KASP SNP set (Figure S3). In other words, allele frequency differences were higher among species when genotyped with MassARRAY and higher within species when genotyped with KASP. This might be a direct consequence of the way how the loci of the different SNP sets were identified. MassARRAY SNPs were selected from whole‐genome data (millions of SNPs) and concentrated on near‐diagnostic markers (i.e., with nearly‐fixed alleles for the investigated species, Kremer et al. 2024), whereas for the KASP SNP set, a limited amount of exonic SNPs (around 3500) was available for selection (Reutimann et al. 2020), limiting chances of identifying near‐diagnostic markers. Similarly, PCAs showed higher discriminatory power with the MassARRAY SNP set than with the KASP SNP set, irrespective of the reference sample set analysed (Figure 2). It is important to emphasize that the SNP sets were developed for species‐discrimination and should therefore not be used for general estimates of neutral genetic parameters like heterozygosity or differentiation within species.

However, the MassARRAY SNP set did not show higher discriminatory power assigning the trees with structure. Here, reference samples were generally identified as purer when analysed with their ‘own’ SNP set (Figure 1), which indicates ascertainment bias. This ascertainment bias can arise from two sources: (i) the samples used for the discovery of the SNPs set and (ii) the reference samples used in the analyses presented here. Despite this, the high discriminatory power in assigning individuals using structure shows that this method can reliably assign individuals even when allele frequency differences are modest, as is the case for the KASP SNP set. Overall, differences among the assignment approaches were small, and no trees were assigned to any species other than their a priori classification. Only a few putatively pure trees were classified as admixed (Figure S2). The difference in the number of markers of the two SNP sets is unlikely to lead to incongruencies in species assignments. Subsampling analyses of the 58 KASP SNPs showed that the accuracy of species assignment is still at least 95% with the 26 most discriminating and with 38 randomly selected markers (Reutimann et al. 2020). We cannot exclude that the three most extreme cases of assignment discrepancy (three individuals identified as pure PET using MassARRAY but showing predominantly ROB ancestry with KASP) represent errors introduced in the laboratory (label/sample switch). Such errors are more likely when analyses are conducted in two independent laboratories. Although difficult to verify retrospectively, these errors appear to be very rare, if they occurred at all.

### 4.2 Assignment Congruency in F1 Hybrids and a Mixed Natural Population

The assignment of F1 hybrids from controlled crosses resulted in assignment probabilities close to the expected 50:50 ratio, confirming that both methods can yield accurate estimates of admixture levels (Figure 3a,c). The reason for the slightly better performance in identifying F1 hybrids of MassARRAY (with INRAE reference samples) likely stems again from ascertainment bias. First, the parental trees of the crosses, except for PET, originated from the southwest of France, i.e., the same part of the range as the reference trees included in the MassARRAY design. Second, the consistently higher proportion of PUB in WSL/KASP compared to INRAE/MassARRAY might derive from the fact that some reference/training individuals for PUB of WSL might be slightly introgressed by PET, overestimating the ancestry of PUB test samples. This interpretation is also supported by increased *H*_e_ in WSL PUB reference samples compared to INRAE PUB reference samples when genotyped with the KASP SNP set (Figure S3). Indeed, it has been suggested that levels of introgression in PUB increase towards more northern latitudes of its distribution range (Chybicki et al. 2012; Franjic et al. 2006; Müller 1999) or, in other words, that introgression of PUB with PET is a prominent feature of populations north of the Alps (and the inner‐Alpine valley of Valais) since they often occur in sympatry with PET (Kremer et al. 2024; Neophytou et al. 2015, 2024; Pütz et al. 2025). Analyses of Reutimann et al. (2020) of the WSL reference samples used here also showed that—though southern and northern PUB clearly form one genetic group—northern PUB seemed slightly less differentiated from PET. These findings highlight the impact of reference individuals in discovery panels for screening discriminatory markers, especially for species with broad distributions and varying admixture levels across their distribution range.

In contrast to the F1 hybrids, we found rather low congruency in estimates of admixture levels between the two SNP sets when analysing natural samples from ISS Valais, an inner‐alpine valley in Switzerland (Figure 4a,b, Figures S4 and S5). Different reasons might explain this finding: First, taxonomic variation in this population is low, maybe too low for being able to demonstrate congruency using a linear regression. In other words, the presence of taxonomically more pure individuals might have increased the agreement between the two genotyping methods. Second, the phylogeographic history of these populations might complicate the analyses. Oak populations from Valais might have a unique evolutionary history, with PUB colonizing the valley from two different refugia (Mátyás and Sperisen 2001; Müller 1999). Some PUB populations may have undergone introgression with PET during the colonization process. Such hybrid swarm populations could represent distinct taxonomic entities and may not exhibit the taxonomic signals typically observed in, for example, F1 crosses. However, further research would be necessary to elucidate the evolutionary history of white oaks in Central Europe to better understand their taxonomic landscape (but see Pütz et al. 2025) and to assess the adaptive relevance and dynamics of introgression (Lazic et al. 2021). Finally, if levels of introgression in the reference samples used for the establishment of each marker set differ, and if different genomic regions are involved in introgression among the two reference sample sets, high congruency would be expected for pure individuals, but not necessarily for highly admixed trees. Overall, our findings highlight the taxonomic complexity of white oaks in specific geographic areas.

### 4.3 Assignment of Additional White Oak Species

Another important finding of this study is that both SNP sets were able to assign additional taxa that they were not initially designed for (Figure 5). Both SNP sets correctly assigned FRA to a separate cluster in structure analyses. Additionally, KASP SNPs were able to identify reference samples of PYR (the MassARRAY SNPs were originally developed to distinguish PYR). This result shows that the erroneous inclusion of additional white oak species (in the test set) due to misidentification in the field should have no consequences on the assignment of the target species, given that higher hierarchical genetic structure is considered (Hausdorf 2025). For example, the KASP SNP set identified PYR only at *K* ≥ 6, whereas these samples would have been identified as PUB when only looking at *K* = 3 (Figure S6). Similarly, FRA samples would have been assigned to PUB when only considering *K* = 4 with the MassARRAY SNPs (Figure S7). Our findings also point to a potential application of these SNP sets to these additional white oak species. However, we acknowledge that the selection of the populations and samples of these additional species was rather arbitrary and opportunistic and does therefore, except for PYR, not present a targeted collection of pure reference samples.

We further tested the diagnostic potential of the KASP SNP set for identifying F1 hybrids of ROB, PET, PUB, and PYR and assigning a mixed natural population of ROB and PYR (ISS Landes), i.e., applying a SNP set that did not include PYR during its development. Here, the KASP SNP set generally failed to correctly assign F1 hybrids involving PYR (Figure 3b,d). However, it correctly identified this additional species in the natural ISS Landes population (Figure 4c,d), which consisted of mainly pure specimens of ROB and PYR. This finding clearly shows the limitations of applying such SNP sets to additional species; whereas identifying pure trees led to accurate and congruent assignment, the inclusion of admixed individuals led to complex outcomes that must be treated with caution. The ability of identifying additional white oak species allows the extension of species identification and, in some cases, assessment of admixture in forest stands, contributing to sustainable forestry (Marshall et al. 2014).

Within the white oak species complex studied here, various putative subspecies have been described, especially in southern and south‐eastern Europe (e.g., Brullo et al. 1999; Mossa et al. 1999). The taxonomic classification of these subspecies is mainly based on morphological and ecological traits, but genetic studies mostly did not find evidence to support a taxonomic discrimination (e.g., Di Pietro et al. 2021; Enescu et al. 2013). This circumstance could have several reasons; (a) the applied marker sets (mostly a limited number of nSSRs) did not have sufficient resolution to reveal small taxonomic differences; (b) the studies establishing the marker panels did not include reference samples from certain subspecies, or (c) there are indeed no or only very small genetic differences among the putative subspecies. Therefore, SNP sets like the ones presented here could contribute to resolving such taxonomic questions. However, although our study included several populations from the putative subspecies, we could not find any evidence for delineation of additional subspecies (results not shown). The SNP sets used in our study were developed for optimal distinction of the three *Quercus* taxa *sensu lato*, hence they are likely not suited for distinguishing such subspecies. Further analyses using whole‐genome sequencing and an adequate inclusion of such putative subspecies are underway (https://www.bfw.gv.at/en/hybrid‐oaks‐genomics/) and will hopefully provide the necessary resolution to shed light on the taxonomical landscape of European white oaks.

## 5 Conclusions and Implications

Extensive admixture and evolutionary entities that may be in the process of diverging into new evolutionary lineages or species can blur species boundaries and introduce inherent uncertainty in the taxonomic assignment of individuals, an essential foundation for biodiversity research and the sustainable management of biological resources (Mace 2004). The availability of two independently developed discriminatory SNP sets for European white oak species offers a unique opportunity to compare molecular species assignment approaches in the same species complex. Our analysis, based on hundreds of white oak samples from across Europe, provides important insights for both scientific and applied uses of species‐discriminatory SNP sets in any (hybridizing) species complex, including wild (e.g., threatened) species and their cultivated/domesticated close relatives. First, ascertainment bias stemming from the selection of reference individuals and markers appear to be largely unavoidable (Geibel et al. 2021). To mitigate ascertainment bias, the development of species‐discriminatory SNP sets should include a geographically broad and representative selection of reference samples as well as a large discovery panel of markers. For example, (refugial) oak populations from southern Europe potentially harbour unique genetic diversity that might currently be underrepresented in the reference sets. However, such excessive coverage of taxon‐wide occurrence and genetic variation is barely feasible in non‐model species. Alternatively, conclusions drawn for samples outside the initial study range should be treated with caution, also regarding the potential identification of putative subspecies described above. Having two complementary SNP sets at hand may highlight inconsistency and help to avoid premature conclusions about species identity and admixture levels. Second, the validation of SNP sets and the choice of assignment approaches ideally include controlled crosses with known ancestry, providing a reliable benchmark for accuracy. Such an approach may not be feasible in all cases, in particular in animals. Third, unintended inclusion of additional or cryptic taxa within the species complex likely requires analyses at higher hierarchy of genetic structure than originally anticipated (Hausdorf 2025). If these aspects are carefully addressed, such SNP sets can substantially advance taxonomic assessments, biodiversity conservation, species management, and ecological research in (hybridizing) species complexes.

## Author Contributions

C.R., A.K., and F.G. acquired funding; C.R., A.K., and O.R. designed the study; C.R. and O.R. performed field work; O.R. and E.G. performed lab work; C.R. and O.R. performed the analyses; C.R. wrote the manuscript with input from all co‐authors.

## Funding

This work was funded by the EvolTree Opportunity grant awarded to Christian Rellstab. The funding body had no role in the study design, data collection, analysis, decision to publish, or preparation of the manuscript.

## Ethics Statement

Plant material was collected from naturally occurring populations (except for artificial crosses) or received from collaborators that used the samples in previous, published studies. Sampling was conducted in accordance with applicable national and local regulations, and the necessary permissions were obtained where required.

## Conflicts of Interest

The authors declare no conflicts of interest.

## Supporting information


**Table S1:** Characteristics of the two species‐discriminatory SNPs sets. ROB = *Q. robur* , PET = *Q. petraea* , PUB = *Q. pubescens*, PYR = *Q. pyrenaica*. ANOVA = Analysis of variance, FAMD = factor analysis for mixed data, LDA = linear discriminant analysis.
**Table S2:** List of near‐diagnostic SNP markers used for MassARRAY genotyping.
**Table S3:** Overview of genotyped samples. Geographic position (averaged per population) and number of samples included in the analyses, grouped by sample type (columns), country and population (rows). Artificial crosses (*n* = 12) excluded. ROB = *Q. robur* , PET = *Q. petraea* , PUB = *Q. pubescens*, PYR = *Q. pyrenaica*, CAN = *Q. canariensis*, FAG = *Q. faginea*, FRA = *Q. frainetto* .
**Table S4:** Overview of the sample sets included in the analyses of reference samples, controlled crosses and a mixed natural population. ROB = *Q. robur* , PET = *Q. petraea* , PUB = *Q. pubescens*, ISS Valais = natural population of PET/PUB, Controlled crosses = F1 hybrids.
**Table S5:** Overview of the sample sets included in the analyses of additional taxa. ROB = *Q. robur* , PET = *Q. petraea* , PUB = *Q. pubescens*, PYR = *Q. pyrenaica*, CAN = *Q. canariensis*, FAG = *Q. faginea*, FRA = *Q. frainetto* . *The reference samples of PYR in WSL/KASP are identical to those for MassARRAY.
**Figure S1:** Congruency of mean *Q*‐values resulting from the different SNP marker sets analysing the WSL (top) and INRAE (bottom) reference samples with *K* = 3. The dotted line corresponds to the 1:1 line. ROB = *Q. robur* , PET = *Q. petraea* , PUB = *Q. pubescens*.
**Figure S2:** Assignment of the original reference individuals (in rows) into ‘pure’ (max. mean *Q*‐value ≥ 0.9 for the target species) or admixed individuals (ADM, mean *Q*‐value < 0.9). Left: WSL samples genotyped with MassARRAY. Right: INRAE samples genotyped with KASP. Numbers refer to percentages of the original reference samples, with a gradient from grey (low) to yellow (high). ROB = *Q. robur* , PET = *Q. petraea* , PUB = *Q. pubescens*.
**Figure S3:** Genetic differentiation and diversity. Above: Genetic differentiation (net nucleotide distance, D, with a gradient from grey [low] to yellow [high]) among the identified clusters, representing species. Lower left corners of the matrices represent values for KASP, upper right values for MassARRAY. Below: Genetic diversity (expected heterozygosity, H_e_) within the identified clusters/species, both for KASP (black) and MassARRAY (grey).
**Figure S4:** Comparison of taxonomic assignment in the ISS Valais population using principal component analyses (PCAs). Comparison (linear regression of second PCs, right) of KASP genotyping (left) and MassARRAY genotyping (middle) using different reference sample sets: All own reference samples (top row), overlap of WSL reference samples (second row), overlap of INRAE reference samples (third row), and overlap of both reference sample sets (bottom row). ROB = *Q. robur* , PET = *Q. petraea* , PUB = *Q. pubescens*, ISS = mixed PET/PUB population of ISS Valais.
**Figure S5:** Comparison of taxonomic assignment in the ISS Valais population using principal component analyses (PCAs). As in Figure S4, but without ROB reference samples. Comparison (linear regression of first PCs, right) of KASP genotyping (left) and MassARRAY genotyping (middle) using different reference samples sets: All own reference samples (top row), overlap of WSL reference samples (second row), overlap of INRAE reference samples (third row), and overlap of both reference sample sets (bottom row). ROB = *Q. robur* , PET = *Q. petraea* , PUB = *Q. pubescens*, ISS = mixed PET/PUB population of ISS Valais.
**Figure S6:** Bar plots from *K* = 2 to *K* = 10 for the KASP reference samples and additional taxa using the KASP SNP set with WSL reference samples (except for PYR). ROB = *Q. robur* , PET = *Q. petraea* , PUB = *Q. pubescens*, PYR = *Q. pyrenaica*, CAN = *Q. canariensis*, FAG = *Q. faginea*, FRA = *Q. frainetto* .
**Figure S7:** Bar plots from *K* = 2 to *K* = 10 for the INRAE reference samples and additional taxa using the MassARRAY SNP set with INRAE reference samples. ROB = *Q. robur* , PET = *Q. petraea* , PUB = *Q. pubescens*, PYR = *Q. pyrenaica*, CAN = *Q. canariensis*, FAG = *Q. faginea*, FRA = *Q. frainetto* .

## Data Availability

Genotype tables and sample metadata can be found on Dryad under https://doi.org/10.5061/dryad.xpnvx0kz0. R‐scripts for analyses and figures are stored on GitLab: https://gitlabext.wsl.ch/rellstab/oakid2‐manuscript. The research described in the publication complies with relevant national laws implementing the Convention on Biological Diversity and Nagoya Protocol agreements. Benefits from this research accrue from the sharing of our data and results on public databases as described above.
