# Supplementary material for: A Comparison of Two Species‐Discriminatory SNP Sets for Taxonomic Assignment in Hybridizing European White Oaks (Quercus sect. Quercus)
Source: Mol Ecol Resour. 2026 Oct 29;26(8):e70221. doi: 10.1111/1755-0998.70221 (PMC13720219; doi:10.1111/1755-0998.70221)
Supplement: Supplementary file 1 [file MEN-26-e70221-s001.docx]

**Supporting Information**

for the manuscript

**A comparison of two species-discriminatory SNP sets for taxonomic assignment in hybridizing European white oaks (*Quercus* sect. *Quercus*)**

by

Christian Rellstab, Erwan Guichoux, Felix Gugerli, Antoine Kremer & Oliver Reutimann

**Supporting Tables**

***Table S1: Characteristics of the two species-discriminatory SNPs sets.*** *ROB = Q. robur, PET = Q. petraea, PUB = Q. pubescens, PYR = Q. pyrenaica. FAMD = Factor analysis for mixed data, LDA = Linear discriminant analysis, ANOVA = Analysis of variance.*

| **SNP set** | **Reference for technology** | **Reference for SNP set development** | **Place of development** | **Genomic resources** | **Species included** | **Discovery panel** | **Discovery method** | **Validation set** | **Final SNP set** |
| --- | --- | --- | --- | --- | --- | --- | --- | --- | --- |
| **KASP** | Semagn, Babu, Hearne & Olsen (2014) | Reutimann, Gugerli & Rellstab (2020) | **WSL** (Swiss Federal Institute for Forest, Snow and Landscape Research) | 3'576 SNPs from pooled amplicon sequencing | ROB, PET, PUB | 71 populations from Switzerland  16 presumably pure European reference individuals | FAMD, LDA, ANOVA, private alleles | 827 individuals reduced to 194 pure reference individuals | 58 SNPs |
| **MassARRAY** | Jurinke, van den Boom, Cantor & Köster (2002) | Kremer et al. (2024) | **INRAE** ([French] National Research Institute for Agriculture, Food and Environment) | a) Whole-genome pooled-sequencing  b) Targeted sequence capture | a) ROB, PET, PUB,  PYR  b) ROB, PET | a) 20 individuals pooled per species from Southwestern France  b) One mixed ROB/PET population from Northern France | Allele frequency differences, *F*_ST_, diagnosticity index | 977 individuals from the Western part of the distribution (24 populations) | 38 SNPs  (7-12 per species) |

***Table S2: List of near-diagnostic SNP markers used for MassARRAY genotyping.***

*For more information, see Kremer et al. (2024).*

| **SNP ID** | **SNP set** |
| --- | --- |
| Sc0000006_2873224 | final |
| Sc0000013_2578823 | final |
| Sc0000014_2037045 | final |
| Sc0000043_1651618 | final |
| Sc0000047_2398879 | final |
| Sc0000053_1344456 | candidate |
| Sc0000053_1639108 | final |
| Sc0000053_1895655 | candidate |
| Sc0000055_2262067 | candidate |
| Sc0000062_118505 | candidate |
| Sc0000083_147504 | final |
| Sc0000085_73024 | final |
| Sc0000088_1796044 | final |
| Sc0000099_1839376 | final |
| Sc0000109_800763 | final |
| Sc0000118_1466708 | final |
| Sc0000135_261350 | final |
| Sc0000145_700044 | final |
| Sc0000158_462639 | final |
| Sc0000170_630013 | final |
| Sc0000192_329301 | final |
| Sc0000203_689887 | final |
| Sc0000203_707735 | final |
| Sc0000228_1091905 | final |
| Sc0000240_289656 | candidate |
| Sc0000269_924931 | final |
| Sc0000274_909817 | final |
| Sc0000307_852597 | final |
| Sc0000339_4638 | final |
| Sc0000381_206331 | final |
| Sc0000447_521057 | final |
| Sc0000481_343721 | final |
| Sc0000517_258593 | final |
| Sc0000517_383812 | final |
| Sc0000695_225347 | final |
| Sc0000778_61930 | final |
| Sc0000796_82698 | final |
| Sc0000967_33996 | final |

***Table S3: Overview of genotyped samples****. Geographic position (averaged per population) and number of samples included in the analyses, grouped by sample type (columns), country and population (rows). Artificial crosses (n=12) excluded. ROB = Q. robur, PET = Q. petraea, PUB = Q. pubescens, PYR = Q. pyrenaica, CAN = Q. canariensis, FAG = Q. faginea, FRA = Q. frainetto.*

| **Country** (Population) | **Latitude** | **Longitude** | **INRAE**  **ROB** | **INRAE**  **PET** | **INRAE**  **PUB** | **INRAE**  **PYR** | **WSL**  **ROB** | **WSL**  **PET** | **WSL**  **PUB** | **ISS**  **Landes** | **ISS**  **Valais** | **CAN** | **FAG** | **FAGxPYR** | **FRA** |
| --- | --- | --- | --- | --- | --- | --- | --- | --- | --- | --- | --- | --- | --- | --- | --- |
| **Algeria** |  |  |  |  |  |  |  |  |  |  |  |  |  |  |  |
| Akfadou Tizi-Ouzou | 36.72180 | 4.45460 |  |  |  |  |  |  |  |  |  | 5 |  |  |  |
| Mechrouba Souk Ahras | 36.38560 | 7.86060 |  |  |  |  |  |  |  |  |  | 5 |  |  |  |
| Ghora Bougous El-Taref | 36.67390 | 8.47020 |  |  |  |  |  |  |  |  |  | 5 |  |  |  |
| Hamza Jijel | 36.74830 | 5.60650 |  |  |  |  |  |  |  |  |  | 6 |  |  |  |
| Chélia Aurès | 35.36790 | 6.63200 |  |  |  |  |  |  |  |  |  |  | 6 |  |  |
| Baloul Saïda | 34.98440 | 0.40410 |  |  |  |  |  |  |  |  |  |  | 6 |  |  |
| Safalou Tiaret | 35.40530 | 1.34860 |  |  |  |  |  |  |  |  |  |  | 6 |  |  |
| **Bosnia Herzegovina** |  |  |  |  |  |  |  |  |  |  |  |  |  |  |  |
| Livno | 44.01500 | 16.63000 | 14 |  |  |  |  |  |  |  |  |  |  |  |  |
| Olovo | 44.15200 | 18.54800 |  | 10 |  |  |  |  |  |  |  |  |  |  |  |
| Ostrožac | 43.68417 | 17.76806 |  |  |  |  |  |  |  |  |  |  |  |  | 13 |
| **France** |  |  |  |  |  |  |  |  |  |  |  |  |  |  |  |
| Arcachon | 44.64938 | -1.19564 |  |  |  |  | 1 |  |  |  |  |  |  |  |  |
| Auros | 44.49200 | -0.14800 |  |  | 7 |  |  |  |  |  |  |  |  |  |  |
| Blaignan | 45.31909 | -0.85642 |  |  |  |  |  |  | 3 |  |  |  |  |  |  |
| Briouant | 43.30600 | 1.04800 | 2 |  |  | 1 |  |  |  |  |  |  |  |  |  |
| Gèdre | 42.78923 | 0.03366 |  |  |  |  |  | 2 |  |  |  |  |  |  |  |
| Landes | 44.23800 | 1.07540 | 1 |  |  | 11 |  |  |  | 16 |  |  |  |  |  |
| Laveyron | 43.97470 | 0.22970 |  | 2 |  |  |  |  |  |  |  |  |  |  |  |
| Orléans | 47.82848 | 1.90941 |  |  |  |  |  | 1 |  |  |  |  |  |  |  |
| Petite Charnie | 48.08590 | -0.16812 | 7 | 7 |  |  | 3 |  |  |  |  |  |  |  |  |
| Tronçais | 46.68000 | 2.82900 |  | 6 |  |  |  |  |  |  |  |  |  |  |  |
| Ventoux | 44.12100 | 5.31200 |  |  | 1 |  |  |  |  |  |  |  |  |  |  |
| **Germany** |  |  |  |  |  |  |  |  |  |  |  |  |  |  |  |
| Grosshansdorf | 53.65000 | 10.25000 |  |  |  |  | 2 |  |  |  |  |  |  |  |  |
| Schädtbek | 54.28333 | 10.26667 |  |  |  |  | 2 |  |  |  |  |  |  |  |  |
| **Great Britain** |  |  |  |  |  |  |  |  |  |  |  |  |  |  |  |
| Roudsea | 54.23332 | -3.02858 |  |  |  |  |  | 3 |  |  |  |  |  |  |  |
| **Greece** |  |  |  |  |  |  |  |  |  |  |  |  |  |  |  |
| Taxiarchis Chalkidiki | 40.42710 | 23.50140 |  |  |  |  |  |  |  |  |  |  |  |  | 15 |
| **Italy** |  |  |  |  |  |  |  |  |  |  |  |  |  |  |  |
| Aspromonte | 38.14300 | 15.93800 |  | 10 |  |  |  |  |  |  |  |  |  |  |  |
| Bosco Pantano | 40.16400 | 16.67100 |  |  | 19 |  |  |  |  |  |  |  |  |  |  |
| Monte Rasu | 40.41667 | 8.99781 |  |  |  |  |  |  | 3 |  |  |  |  |  |  |
| Superano | 40.03417 | 18.26417 |  |  |  |  |  |  |  |  |  |  |  |  | 14 |
| Tatti | 43.35168 | 10.97681 |  |  |  |  |  |  | 1 |  |  |  |  |  |  |
| Torralba | 40.50778 | 8.77056 |  |  |  |  |  |  | 3 |  |  |  |  |  |  |
| **Romania** |  |  |  |  |  |  |  |  |  |  |  |  |  |  |  |
| Hidiselu de Sus | 46.93069 | 22.09375 |  |  |  |  |  |  |  |  |  |  |  |  | 7 |
| Lugoj | 44.71667 | 24.46667 |  |  |  |  |  |  |  |  |  |  |  |  | 15 |
| Slatina | 45.71667 | 21.98333 |  |  |  |  |  |  |  |  |  |  |  |  | 15 |
| **Spain** |  |  |  |  |  |  |  |  |  |  |  |  |  |  |  |
| Alcala de los Gazules | 36.01739 | -5.13178 |  |  |  |  |  |  |  |  |  | 14 |  |  |  |
| Cabañeros | 39.33600 | -4.50200 |  |  |  |  |  |  |  |  |  |  |  | 4 |  |
| Collado de los Jardines | 38.38917 | -3.49250 |  |  |  |  |  |  |  |  |  |  | 3 |  |  |
| Font Roja | 38.75800 | -0.62200 |  |  |  |  |  |  |  |  |  |  | 4 |  |  |
| Hoya Del Nevazo | 36.95700 | -3.42300 |  |  |  | 1 |  |  |  |  |  |  |  |  |  |
| La Calanchera | 39.57200 | -4.64700 |  |  |  | 5 |  |  |  |  |  |  |  |  |  |
| Los Barrios | 36.22539 | -5.58306 |  |  |  |  |  |  |  |  |  | 12 |  |  |  |
| Moncayo | 41.79575 | -1.78665 |  |  |  |  |  | 4 |  |  |  |  |  |  |  |
| Montejo | 41.11700 | -3.50000 |  | 1 |  |  |  |  |  |  |  |  |  |  |  |
| Pedro | 40.07900 | -5.73900 | 2 |  |  | 7 |  |  |  |  |  |  |  |  |  |
| Rascafria | 40.91100 | -3.89800 |  |  |  | 3 |  |  |  |  |  |  |  |  |  |
| Redueña | 40.81331 | -3.60164 |  |  |  |  |  |  |  |  |  |  | 12 |  |  |
| Rio Lobos | 41.72900 | -3.04000 |  |  |  |  |  |  |  |  |  |  | 4 |  |  |
| Talayuela | 39.99900 | -5.69600 |  |  |  |  |  |  |  |  |  |  |  | 3 |  |
| Venturada | 40.80192 | -3.60903 |  |  |  |  |  |  |  |  |  |  | 10 |  |  |
| **Sweden** |  |  |  |  |  |  |  |  |  |  |  |  |  |  |  |
| Gålö | 59.09556 | 18.31889 |  |  |  |  | 3 |  |  |  |  |  |  |  |  |
| Nora | 59.57500 | 15.02083 |  |  |  |  | 4 |  |  |  |  |  |  |  |  |
| **Switzerland** |  |  |  |  |  |  |  |  |  |  |  |  |  |  |  |
| Arnex-sur-Nyon | 46.36826 | 6.17966 |  |  |  |  | 4 | 1 |  |  |  |  |  |  |  |
| Au (SG) | 47.43811 | 9.62673 |  |  |  |  |  | 5 |  |  |  |  |  |  |  |
| Ayent | 46.26634 | 7.39800 |  |  |  |  |  |  | 1 |  |  |  |  |  |  |
| Bärschwil | 47.39230 | 7.46411 |  |  |  |  |  |  | 1 |  |  |  |  |  |  |
| Belp | 46.88715 | 7.53190 |  |  |  |  | 2 |  |  |  |  |  |  |  |  |
| Beromünster | 47.24918 | 8.21379 |  |  |  |  | 4 |  |  |  |  |  |  |  |  |
| Bevaix | 46.92141 | 6.80351 |  |  |  |  |  | 3 |  |  |  |  |  |  |  |
| Birmenstorf (AG) | 47.43595 | 8.25529 |  |  |  |  | 3 |  |  |  |  |  |  |  |  |
| Bonfol | 47.47228 | 7.16337 | 3 |  |  |  | 6 |  |  |  |  |  |  |  |  |
| Breggia | 45.85762 | 9.03070 |  |  |  |  |  |  | 3 |  |  |  |  |  |  |
| Brislach | 47.43946 | 7.55117 |  |  |  |  | 2 | 1 |  |  |  |  |  |  |  |
| Buch am Irchel | 47.53564 | 8.61712 |  |  |  |  |  | 2 |  |  |  |  |  |  |  |
| Bulle | 46.61891 | 7.07309 |  |  |  |  | 1 |  |  |  |  |  |  |  |  |
| Büren an der Aare | 47.12392 | 7.35630 |  |  |  |  |  | 3 |  |  |  |  |  |  |  |
| Caslano | 45.96226 | 8.88656 |  |  |  |  |  |  | 3 |  |  |  |  |  |  |
| Centovalli | 46.17936 | 8.68107 |  |  |  |  |  | 4 |  |  |  |  |  |  |  |
| Cevio | 46.34795 | 8.60713 |  |  |  |  |  | 4 |  |  |  |  |  |  |  |
| Champagne | 46.84080 | 6.64308 |  |  |  |  |  | 4 |  |  |  |  |  |  |  |
| Cheyres | 46.81370 | 6.79271 |  |  |  |  |  | 3 |  |  |  |  |  |  |  |
| Cureglia | 46.04205 | 8.94982 |  |  |  |  | 4 |  |  |  |  |  |  |  |  |
| Domat/Ems | 46.81902 | 9.43212 |  |  |  |  |  | 1 |  |  |  |  |  |  |  |
| Eiken | 47.53587 | 7.99342 |  |  |  |  | 3 |  |  |  |  |  |  |  |  |
| Genolier | 46.44123 | 6.23088 |  |  |  |  |  |  | 1 |  |  |  |  |  |  |
| Gordola | 46.19545 | 8.86323 |  |  |  |  |  |  | 3 |  |  |  |  |  |  |
| Grono | 46.25046 | 9.13591 |  |  |  |  |  | 4 |  |  |  |  |  |  |  |
| Heimiswil | 47.06778 | 7.69535 |  |  |  |  | 4 |  |  |  |  |  |  |  |  |
| Hofstetten-Flüh | 47.48307 | 7.50329 |  |  |  |  |  |  | 1 |  |  |  |  |  |  |
| Kaltbrunn | 47.20296 | 9.01742 |  |  |  |  | 5 |  |  |  |  |  |  |  |  |
| Laufen-Uhwiesen | 47.67352 | 8.63704 |  |  |  |  |  | 3 |  |  |  |  |  |  |  |
| Lugano | 46.01142 | 9.00915 |  |  |  |  |  |  | 4 |  |  |  |  |  |  |
| Maggia | 46.24245 | 8.70248 |  |  |  |  | 3 |  |  |  |  |  |  |  |  |
| Malans | 46.97870 | 9.59195 |  |  |  |  |  | 1 |  |  |  |  |  |  |  |
| Merishausen | 47.76073 | 8.61594 |  |  |  |  |  |  | 1 |  |  |  |  |  |  |
| Mesocco | 46.37974 | 9.22648 |  |  |  |  |  | 4 |  |  |  |  |  |  |  |
| Mezzovico-Vira | 46.08612 | 8.91074 |  |  |  |  |  |  | 4 |  |  |  |  |  |  |
| Mont-sur-Rolle | 46.48046 | 6.33151 |  |  |  |  |  |  | 2 |  |  |  |  |  |  |
| Morcote | 45.94387 | 8.90688 |  |  |  |  |  | 4 |  |  |  |  |  |  |  |
| Muhen | 47.32665 | 8.07097 |  |  |  |  |  | 4 |  |  |  |  |  |  |  |
| Neunkirch | 47.68111 | 8.52656 |  |  |  |  |  |  | 1 |  |  |  |  |  |  |
| Rapperswil-Jona | 47.23921 | 8.83944 | 3 |  |  |  | 4 |  |  |  |  |  |  |  |  |
| Remigen | 47.51860 | 8.16267 |  |  |  |  |  |  | 3 |  |  |  |  |  |  |
| Rheinfelden | 47.53697 | 7.78498 |  |  |  |  | 5 |  |  |  |  |  |  |  |  |
| Romainmôtier-Envy | 46.69639 | 6.47176 |  |  |  |  |  |  | 2 |  |  |  |  |  |  |
| Romanshorn | 47.56247 | 9.33985 |  |  |  |  | 3 |  |  |  |  |  |  |  |  |
| Rueun | 46.78529 | 9.14241 |  |  |  |  |  | 1 |  |  |  |  |  |  |  |
| Sachseln | 46.87635 | 8.25092 |  |  |  |  | 2 |  |  |  |  |  |  |  |  |
| Saillon | 46.17064 | 7.16662 |  |  |  |  |  |  | 4 |  |  |  |  |  |  |
| Salgesch | 46.32029 | 7.58096 |  |  |  |  |  |  | 1 |  |  |  |  |  |  |
| Trin | 46.82798 | 9.36661 |  |  |  |  |  | 3 |  |  |  |  |  |  |  |
| Turtmann | 46.30174 | 7.71565 |  |  |  |  |  |  | 2 |  |  |  |  |  |  |
| Ulmiz | 46.92470 | 7.20603 |  |  |  |  | 4 |  |  |  |  |  |  |  |  |
| Valais | 46.30111 | 7.66661 |  |  |  |  |  |  |  |  | 34 |  |  |  |  |
| Verdabbio | 46.29056 | 9.17701 |  |  |  |  | 2 |  |  |  |  |  |  |  |  |
| Vétroz | 46.22122 | 7.25516 |  |  |  |  |  |  | 2 |  |  |  |  |  |  |
| Vionnaz | 46.30387 | 6.88096 |  |  |  |  |  | 1 |  |  |  |  |  |  |  |
| **Sum** |  |  | **32** | **36** | **27** | **28** | **76** | **66** | **49** | **16** | **34** | **47** | **51** | **7** | **79** |

***Table S4: Overview of the sample sets included in the analyses of reference samples, controlled crosses and a mixed natural population.***
*ROB = Q. robur, PET = Q. petraea, PUB = Q. pubescens, ISS Valais = natural population of PET/PUB, Controlled crosses = F1 hybrids.*

| **Species** | **Set 1** | **Set 2** | **Set 3** | **Set 4** |
| --- | --- | --- | --- | --- |
| Reference samples | WSL | INRAE | WSL | INRAE |
| SNP set | both | both | KASP | MassARRAY |
| ROB | 49 | 32 | 76 | 32 |
| PET | 44 | 36 | 66 | 36 |
| PUB | 43 | 26 | 49 | 27 |
| ISS Valais | - | - | 34 | 34 |
| Controlled crosses | - | - | 6 | 6 |
| **Total** | **136** | **94** | **240** | **144** |

***Table S5: Overview of the sample sets included in the analyses of additional taxa.***  *ROB = Q. robur, PET = Q. petraea, PUB = Q. pubescens, PYR = Q. pyrenaica, CAN = Q. canariensis, FAG = Q. faginea, FRA = Q. frainetto. *The reference samples of PYR in WSL/KASP are identical to those for MassARRAY.*

| **Species** | **Set 5** | **Set 6** | **Set 7** | **Set 8** |
| --- | --- | --- | --- | --- |
| Reference samples | WSL | INRAE | WSL | INRAE |
| SNP set | KASP | MassARRAY | KASP | MassARRAY |
| ROB | 76 | 32 | 76 | 32 |
| PET | 66 | 36 | 66 | 36 |
| PUB | 49 | 27 | 49 | 27 |
| PYR | 28* | 28 | 28* | 28 |
| CAN | 47 | 16 | - | - |
| FAG | 51 | 25 | - | - |
| FAG×PYR | 7 | 1 | - | - |
| FRA | 79 | 26 | - | - |
| ISS Landes | - | - | 16 | 16 |
| Controlled crosses | - | - | 12 | 12 |
| **Total** | **403** | **191** | **247** | **151** |

**Supporting Figures**

***Figure S1: Congruency of mean Q-values resulting from the different SNP marker sets*** *analyzing the WSL (top) and INRAE (bottom) reference samples with K=3. The dotted line corresponds to the 1:1 line. ROB = Q. robur, PET = Q. petraea, PUB = Q. pubescens.*

***Figure S2: Assignment of the original reference individuals*** *(in rows) into "pure" (max. mean Q-value ≥ 0.9 for the target species) or admixed individuals (ADM, mean Q-value < 0.9). Left: WSL samples genotyped with MassARRAY. Right: INRAE samples genotyped with KASP. Numbers refer to percentages of the original reference samples, with a gradient from grey (low) to yellow (high). ROB = Q. robur, PET = Q. petraea, PUB = Q. pubescens.*

***Figure S3: Genetic differentiation and diversity****. Above: Genetic differentiation (net nucleotide distance, D, with a gradient from grey [low] to yellow [high]) among the identified clusters, representing species. Lower left corners of the matrices represent values for KASP, upper right values for MassARRAY. Below: Genetic diversity (expected heterozygosity, H_e_) within the identified clusters/species, both for KASP (black) and MassARRAY (grey).*

***Figure S4:*** ***Comparison of*** ***taxonomic assignment in the ISS Valais population using principal component analyses (PCAs)****. Comparison (linear regression of second PCs, right) of KASP genotyping (left) and MassARRAY genotyping (middle) using different reference sample sets: All own reference samples (top row), overlap of WSL reference samples (second row), overlap of INRAE reference samples (third row), and overlap of both reference sample sets (bottom row). ROB = Q. robur, PET = Q. petraea, PUB = Q. pubescens, ISS = mixed PET/PUB population of ISS Valais.*

***Figure S5:*** ***Comparison of*** ***taxonomic assignment in the ISS Valais population using principal component analyses (PCAs)****. As in Figure S4, but without ROB reference samples. Comparison (linear regression of first PCs, right) of KASP genotyping (left) and MassARRAY genotyping (middle) using different reference samples sets: All own reference samples (top row), overlap of WSL reference samples (second row), overlap of INRAE reference samples (third row), and overlap of both reference sample sets (bottom row). ROB = Q. robur, PET = Q. petraea, PUB = Q. pubescens, ISS = mixed PET/PUB population of ISS Valais.*

***Figure S6:*** *Bar plots from K=2 to K=10 for the KASP reference samples and additional taxa using the KASP SNP set with WSL reference samples (except for PYR). ROB = Q. robur, PET = Q. petraea, PUB = Q. pubescens, PYR = Q. pyrenaica, CAN = Q. canariensis, FAG = Q. faginea, FRA = Q. frainetto.*

***Figure S7:*** *Bar plots from K=2 to K=10 for the INRAE reference samples and additional taxa using the MassARRAY SNP set with INRAE reference samples. ROB = Q. robur, PET = Q. petraea, PUB = Q. pubescens, PYR = Q. pyrenaica, CAN = Q. canariensis, FAG = Q. faginea, FRA = Q. frainetto.*

**References**

Jurinke, C., van den Boom, D., Cantor, C. R. & Köster, H. (2002). The Use of MassARRAY Technology for High Throughput Genotyping. In: J. Hoheisel, A. Brazma, K. Büssow*, et al.* (Eds.), *Chip Technology*, pp. 57-74, Berlin, Heidelberg. Springer Berlin Heidelberg.

Kremer, A., Delcamp, A., Lesur, I., Wagner, S., Rellstab, C., Guichoux, E. & Leroy, T. (2024). Whole-genome screening for near-diagnostic genetic markers for four western European white oak species identification. *Annals of Forest Science*, *81*, 21.

Reutimann, O., Gugerli, F. & Rellstab, C. (2020). A species-discriminatory single-nucleotide polymorphism set reveals maintenance of species integrity in hybridizing European white oaks (*Quercus* spp.) despite high levels of admixture. *Annals of Botany*, *125*, 663-676.

Semagn, K., Babu, R., Hearne, S. & Olsen, M. (2014). Single nucleotide polymorphism genotyping using Kompetitive Allele Specific PCR (KASP): overview of the technology and its application in crop improvement. *Molecular Breeding*, *33*, 1-14.
